# Does nicotinic acid (niacin) lower blood pressure?

**DOI:** 10.1111/j.1742-1241.2008.01934.x

**Published:** 2009-01

**Authors:** H E Bays, D J Rader

**Affiliations:** 1 Louisville Metabolic and Atherosclerosis Research CenterLouisville, KY, USA; 2 Cardiovascular Metabolism Unit, Institute for Diabetes, Obesity, and MetabolismPhiladelphia, PA, USA

## Abstract

Nicotinic acid (niacin) is a well-established treatment for dyslipidaemia – an important cardiovascular disease (CVD) risk factor. However, niacin may also reduce blood pressure (BP), which is another important CVD risk factor. This review examines the limited publicly available data on niacin’s BP effects. Acute administration of immediate-release niacin may lower BP because of niacin’s acute vasodilatory effects. Although not always supported by clinical trial data, the package insert of a prescription, extended-release niacin describes niacin-induced acute hypotension. From a chronic standpoint, larger studies, such as the Coronary Drug Project, suggest that niacin may lower BP when administered over a longer period of time. *Post hoc* analyses of some of the more recent niacin clinical trials also support a more chronic, dose-dependent, BP-lowering effect of niacin. Because laropiprant [a prostaglandin D_2_ (PGD_2_) type 1 (DP1) receptor antagonist] does not attenuate niacin’s BP-lowering effects, it is unlikely that any chronic lowering of BP by niacin is due to dilation of dermal vessels through activation of the DP1 receptor by PGD_2._ Further research is warranted to evaluate the extent and mechanisms of niacin’s effects on BP.

**Review Criteria**

The information collected for this review included a literature search using key terms. The trials selected and described in this review were those that contained data describing nicotinic acid’s acute and chronic effects upon blood pressure.

**Message for the Clinic**

Nicotinic acid is a well-known lipid-altering drug. The improved cardiovascular outcomes with nicotinic acid therapy are thought to be related to its favourable lipid effects. However, less known is that nicotinic acid may also lower blood pressure, particularly at higher doses administered over a longer period of time. It is possible that some of nicotinic acid’s cardiovascular outcome benefits may be related to an improvement in high blood pressure, which is a major cardiovascular disease risk factor.

## Introduction

Nicotinic acid (niacin) is a lipid-altering therapy used since the 1950s (1) that effectively raises high-density lipoprotein cholesterol (HDL-C) levels. Niacin also reduces triglyceride (TG), and particularly at higher doses, significantly lowers low-density lipoprotein cholesterol levels (2,3). The clinical trial data support niacin as a therapeutic agent that reduces the risk of atherosclerotic coronary heart disease (CHD) events and reduces atherosclerotic progression in patients with CHD and/or other cardiovascular risk factors (4–18). Although the favourable lipid effects of niacin have been known for decades, it is less recognised that niacin may also favourably lower blood pressure (BP).

The major adverse experience limiting the more widespread use of niacin is cutaneous vasodilation resulting in flushing (19–21). Other vasoactive properties of niacin include possible improvements in vascular headaches (22). The package insert for certain niacin prescription formulations describe rare cases of acute syncope, hypotension, and postural hypotension, especially when co-administered with ganglionic-blocking and vasoactive medications (23,24). However, there is a paucity of peer-reviewed published literature regarding these acute effects and potential drug interactions, and published data regarding niacin’s chronic BP effects are limited.

## Methods

The literature supporting this review was derived from an English-language search of PubMed using the title words ‘niacin’ or ‘nicotinic acid’ along with various Medical Subject Heading (MeSH) terms (in various combinations), including ‘adult’, ‘blood’, ‘blood pressure’, ‘blood pressure monitoring, ambulatory’, ‘blood supply’, ‘cardiac output’, ‘catecholamines’, ‘epinephrine’, ‘heart rate’, ‘hemodynamic processes’, ‘human’, ‘hypertension’, ‘hypotension’, ‘norepinephrine’, ‘randomized controlled trials’, ‘stroke volume’, ‘vascular resistance’ and ‘vasodilator agents’. Additional references were obtained from treatment guidelines and other English-language publications involving major clinical outcome reviews and angiographic or ultrasonographic studies that included reported mean BP at baseline and at end-point. Case reports and case studies were excluded.

## Results

### Acute effects of niacin on blood pressure and related haemodynamic parameters

Two illustrative published studies specifically evaluated and reported the short-term effects of niacin on BP (25,26). In one parallel-group study, BP and other haemodynamic parameters [e.g. waveform data on stroke volume (SV), cardiac output (CO) and systemic vascular resistance (SVR)] were measured at 0, 15 and 30 min during a 30-min baseline period, followed by niacin infusion at 2.8 mg/min for up to 60 min. After niacin infusion, this study evaluated similar BP and haemodynamic measurements at 30, 45 and 60 min in 11 normotensive and 10 hypertensive subjects (stage 1 hypertension) (25). Intravenous niacin infusion (2.8 mg/min; ∼0.04 mg/kg/min) had no significant effects on BP in normotensive individuals (*n*= 11; average seated BP ≤ 130/80 mmHg) (25). CO (determined by pulse waveform analysis) was unaffected by niacin infusion at 30 and 60 min in these subjects; however, mean heart rate (HR) increased by 12% to 13%, from 59 beats/minute (bpm) at baseline to 66 to 67 bpm at 30 to 60 min (p < 0.01). This chronotropic effect of niacin in normotensives was associated with significant acute declines in (i) SVR 60 min after niacin infusion, (ii) SV at 30 min, (iii) overall vascular compliance [expressed as the SV/pulse pressure (PP) ratio] at 30 and 60 min and (iv) the mean large-artery elasticity index at 30 and 60 min (Table 1) (25). Small-artery elasticity did not undergo significant change.

**Table 1:** Acute haemodynamic effects of niacin infusion in normotensive and hypertensive subjects

	Normotensives (*n*= 11)			Hypertensives (*n*= 10)		
Haemodynamic parameter (mean ± SD)	Baseline	30 min	60 min	Baseline	30 min	60 min
Systolic BP, mmHg	109 ± 3	109 ± 2	108 ± 2	136 ± 4	130 ± 3†§	129 ± 3†§
Diastolic BP, mmHg	65 ± 2	62 ± 2	63 ± 2	89 ± 2	84 ± 3*§	85 ± 3*§
Mean BP, mmHg	79 ± 2	78 ± 2	78 ± 2	105 ± 2	99 ± 2†§	100 ± 3†§
Pulse pressure, mmHg	44 ± 2	46 ± 2	45 ± 2	47 ± 2	46 ± 2	44 ± 2*
Heart rate, beats/min	59 ± 2	67 ± 3†	66 ± 3†	71 ± 3§	81 ± 4†‡	79 ± 4†‡
Stroke volume, ml/beat	91 ± 3	85 ± 5*	86 ± 4	88 ± 5	79 ± 6*	80 ± 6*
Cardiac output, l/min	5.4 ± 0.2	5.5 ± 0.2	5.5 ± 0.15	6.0 ± 0.2	6.0 ± 3	5.9 ± 0.3
Systemic vascular resistance, dynes/s/cm^5^	1215 ± 44	1156 ± 45	1145 ± 41*	1492 ± 93‡	1515 ± 182	1408 ± 99*‡
SV/PP (overall compliance), ml/mmHg	2.12 ± 0.09	1.84 ± 0.11†	1.93 ± 0.09*	1.91 ± 0.14	1.76 ± 0.19	1.89 ± 0.16
C1 (large-artery elasticity index)	15.8 ± 1.0	13.9 ± 1.0*	14.2 ± 1.0*	11.9 ± 1.0§	11.3 ± 1.4	11.4 ± 1.2
C2 (small-artery elasticity index)	7.5 ± 1.1	7.1 ± 0.5	6.7 ± 1.1	6.5 ± 1.1	5.4 ± 1.1	5.6 ± 1.0

* p ≤ 0.05

† p ≤ 0.01 for comparison with baseline;

‡ p ≤ 0.05

§ p ≤ 0.01 for comparison between hypertensive and normotensive subjects. Reproduced with permission from Gadegbeku et al. (25). BP, blood pressure; SV, stroke volume; PP, pulse pressure.

In contrast, this same study suggested that acute niacin administration may lower BP in patients with hypertension. Those with hypertension experienced significant decreases in systolic BP (SBP), diastolic BP (DBP), mean arterial pressure (MAP), PP, SVR and SV from baseline at up to 60 min after the onset of niacin infusion (Table 1) (25). Significant decreases from baseline were observed in (i) mean SBP by a maximum of 7 mmHg (5%), (ii) DBP by a maximum of 4 mmHg (4%), (iii) MAP by a maximum of 6 mmHg (6%), (iv) PP by a maximum of 3 mmHg (6%), (v) SVR by a maximum of 84 dynes/s/cm^5^ (6%) at 60 min after an initial increase and (vi) SV by a maximum of 9 ml/beat (10%; each p ≤ 0.05 vs. baseline). HR increased significantly in patients with hypertension, by a similar proportion to the effect observed in normotensives [maximum = 10 bpm (11%); p ≤ 0.01 vs. baseline], although patients with hypertension had a significantly higher HR at baseline (71 vs. 59 bpm; p ≤ 0.01). Unlike their normotensive counterparts, patients with hypertension did not experience significant acute declines in overall vascular compliance or the large-artery elasticity index (25).

One proposed mechanism for niacin’s divergent effects on BP in normotensives compared with those with hypertension is differential effects upon large-artery compliance. In normotensive patients, chronotropic responses to niacin infusion were similar to the responses in those with hypertension, but these acute increases in HR did not affect CO in normotensives, possibly because of a countervailing significant decrease in SV. The finding of stable BP despite a significant acute decrease in SVR in normotensives may suggest that peripheral vasodilation triggers counter-regulatory mechanisms, potentially with vasoconstriction in other vascular beds. The significant acute depressor effects of niacin infusion in patients with hypertension may reflect a reduced ability to modulate large-artery compliance. Those with hypertension are known to have decreased vascular compliance and may also have vascular stiffness that is less responsive to changes in counter-regulatory vasoconstrictor hormones (27). The fact that the chronotropic effects of niacin infusion were similar in patients with or without hypertension, but were insufficient to maintain BP in those with hypertension, may also suggest impaired baroreceptor responses in these patients (28). Another mechanism, albeit less likely, for the significant acute depressor effects of niacin in patients with hypertension is an increased prostacyclin response to niacin in the vasculature of these patients compared with their normotensive counterparts (25).

Given that niacin presumably has similar metabolic effects in those with high BP compared with those with normal BP, then niacin-related metabolic effects would not be a plausible explanation for BP differences between hypertensive versus non-hypertensive groups (25,29). However, niacin does affect metabolic parameters often thought to affect BP, such as circulating free fatty acids (FFAs). After acute oral administration of 500 mg of niacin in man, arterial FFA levels decrease within minutes. After about 2 h, FFAs undergo a ‘rebound’ elevation (30). In longer-term trials of chronic oral niacin administration, this ‘rebound’ (21,31–33) has been described to last as long as ≥ 9 h, resulting in increased circulating FFAs (21,31–34). Increased FFAs have been hypothesised to contribute to insulin resistance (26,34), which, in turn, may contribute to high BP (35).

The observation that niacin acutely decreases FFA levels for a few hours might conceivably be consistent with an acute decrease in BP. However, it is unclear if niacin administration increases or decreases total daily FFA release and circulatory exposure. Furthermore, studies suggest that extended-release niacin may have less potential for FFA rebound (vs. immediate-release formulations), especially after several months of use (36). In the previously described study regarding intravenous niacin’s effects upon BP, the haemodynamic responses to niacin in hypertensive vs. normotensive subjects were not ascribed to between-group differences in effects of niacin on FFAs or TG (25). Because the relationship of niacin’s effects on FFAs and BP is uncertain, it seems unlikely that the short-term, acute reduction in FFA would account for acute BP lowering. From a more chronic standpoint, if a rebound increased in FFA and increased in insulin resistance were anticipated to have any BP effect, then it would be expected to raise, not lower, chronic BP (35).

A second illustrative study evaluating the potential relationship between niacin’s effects on FFAs, insulin sensitivity, and BP was a prospective, randomised, double-blind, placebo-controlled crossover study in seven healthy volunteers (26). The active treatment was immediate-release niacin orally administered as one 250-mg capsule twice daily for the first week, followed by two 250-mg capsules twice daily for the second week. Using the hyperinsulinaemic–euglycaemic clamp method, the investigators found that niacin reduced insulin sensitivity compared with placebo and significantly decreased the rate of glucose infusion needed to maintain euglycaemia (26.2 vs. 31.5 μmol/kg/min; p = 0.002). This was attributable to a decline in non-oxidative glucose disposal associated with niacin treatment. Interestingly, with the twice daily dosing regimen, fasting glucose, insulin, FFAs, energy intake and substrate oxidation were unchanged compared with placebo. However, despite the increase in insulin resistance, niacin administration did not significantly affect SBP or DBP compared with placebo (Table 2) (26).

**Table 2:** Acute metabolic and haemodynamic effects of niacin

Parameter (mean ± SEM)	Placebo	Niacin
Glucose infusion rate, μmol/kg FFM/min	41.5 ± 5.8	34.2 ± 6.8*
Non-oxidative glucose disposal, mg/min	314 ± 73	218 ± 66*
Mean 24-h blood pressure (BP), mmHg	82.1 ± 2.0	81.8 ± 3.3
24-h systolic BP, mmHg	112.6 ± 2.3	115.3 ± 4.4
24-h diastolic BP, mmHg	67.1 ± 2.0	65.1 ± 2.8
Forearm blood flow, ml/100 ml/min	4.3 ± 0.3	4.8 ± 0.4
Plasma nitrate, μmol/l	18.4 ± 2.4	17.0 ± 4.2
Urinary nitrate, μmol/l	686 ± 126	705 ± 194
Urinary prostaglandin E_2,_ ng/24 h	287 ± 56	195 ± 42

FFM, fat-free mass.

* p = 0.002. Reproduced with permission from Kelly et al. (26).

Thus the extent to which, and the mechanisms by which short-term niacin administration affects BP remain unclear. However, what is clear is that niacin administration often causes flushing because of marked vasodilation, and up to a 100% increase in cutaneous perfusion and up to a 200% increase in skeletal-muscle blood perfusion (37). Given the significant surface area of skin and the degree of increased cutaneous blood perfusion that can occur with niacin, one might speculate that when niacin does acutely lower BP, in the absence of cardiac dysrhythmias, this may, in part, be due to shunting of blood from large vessels to dilated cutaneous small vessels (and possibly skeletal muscle vessels). Clinically, during whole-body heat stress, active vasodilation is known to account for 85–95% of the overall cutaneous vasodilator response, which may increase cutaneous vascular conductance and contribute to orthostasis (38). It is therefore plausible that vasodilatation might be responsible for the acute BP-lowering effects of niacin in rare patients, although other cardiovascular mechanisms (such as direct effects on the heart and other vessels) cannot be excluded.

### Effects of chronic niacin therapy on blood pressure

Among studies of chronic niacin administration that reported both baseline and on-treatment mean values for SBP and DBP (or changes in BP), most described BP changes from baseline to end-point (or between niacin-treated and control groups) that were not statistically significant (Table 3) (6,8,13,18,39). Although a substantial proportion of subjects in these trials had a history of hypertension and/or used antihypertensive medications (40–70%) (4,18), these trials generally failed to report the use of antihypertensive medications, changes in medication during the study, or incident hypertension (or hypotension) in patients receiving niacin or niacin-containing regimens compared with control groups. Thus, these trials are not optimal in assessing the potential chronic BP-lowering effects of niacin.

**Table 3:** Effects of niacin and niacin-containing regimens on blood pressure in major outcome studies with mean values reported at both baseline and end-point

Study	Patients	Duration; outcome measure	Daily dose of regimen (*n* with available BL and EP data for BP)	Baseline mean BP, mmHg	End-point (or on-trial) mean BP, mmHg	Comparison
**Niacin monotherapy**						
Coronary Drug Project (39)	*n*= 8341 men with previous MI	5–8.5 years; total mortality (also coronary events)	Niacin 3.0 g (*n*= 621)	SBP = 129.7 DBP = 81.7	SBP = 131.7 DBP = 80.2	*z* = −1.40* vs. placebo for SBP *z* = −1.11* vs. placebo for DBP
			Placebo (*n*= 1607) Placebo (*n*= 1606)	SBP = 129.1 DBP = 81.5	SBP = 132.3 DBP = 80.6	See above
**Niacin-containing regimens**						
AFREGS, Whitney et al. (18)	*n*= 143 military retirees ages < 76 years with CHD and low HDL-C	30 months (50 weeks for BP); primary: % change in global angiographic stenosis; secondary: composite end-point (hosp. for angina, MI, TIA and stroke, death, and CV procedures (also angiographic end-points)	Stepped care (*n*= 71): gemfibrozil 1.2 g/day Niacin 0.25–3.0 g/day CME: 2–16 g/day	SBP = 139.0 DBP = 75.3	ΔSBP = −9.8% ΔDBP = 6.8%	SBP p = 0.14 vs. placebo DBP p > 0.2 vs. placebo
			Placebo (*n*= 72)	SBP = 138.9 DBP = 76.3	ΔSBP = −6.6%ΔDBP = 4.3%	
HATS, Brown et al. (8)	*n*= 160 patients with CHD and low HDL-C; 146 patients completed treatment	3 years; arteriographic evidence of Δcoronary stenosis + first CV event (death, MI, stroke, revascularisation)	Niacin 0.5–4 g (mean = 2.4 g) + simvastatin 10–20 mg (mean = 13 mg) (*n*= 33)	SBP/DBP = 124/78	SBP/DBP = 125/77	ns vs. baseline
			Niacin–simvastatin (as above) + antioxidants (800 IU vitamin E, 1 g, vitamin C; 25 mg, β-carotene; 100 μg selenium) (*n*= 40)	SBP/DBP = 130/81	SBP/DBP = 129/79	p < 0.05 vs. baseline
			Placebo (*n*= 34)	SBP/DBP = 125/80	SBP/DBP = 127/80	ns vs. baseline
CLAS, Blankenhorn et al. (6)	*n*= 78 non-smoking men ages 40–59 years with history of CABG	Up to 4 years; common carotid intima-media thickening	Niacin: mean = 4.2 g Colestipol: mean = 30.1 g (*n*= 24)	SBP = 122 DBP = 79	SBP = 118 DBP = 78	BL: p = 0.33 vs. placebo (SBP) p = 0.32 vs. placebo (DBP) EP: p = 0.86 vs. placebo (SBP) p = 0.83 vs. placebo (DBP)
			Placebo (*n*= 22)	SBP = 118 DBP = 77	SBP = 119 DBP = 89	
Stockholm Ischaemic Heart Study, Carlson and Rosenhamer (13)	*n*= 555 consecutive MI survivors ages < 70 years	Up to 5 years; total mortality, CV-specific mortality; non-fatal CV events	Colestipol = 2 g + niacin = 3 g(*n*= 279 BL; *n*= 238, 1 year; *n*= 211, 2 years; *n*= 183, 3 years)	SBP = 133 DBP = 82	1 year: SBP = 148 DBP = 86 2 years: SBP = 149 DBP = 88 3 years: SBP = 148 DBP = 87	p = ns for each comparison vs. control at each time point
			Control (*n*= 276 BL; *n*= 245, 1 year; *n*= 211, 2 years; *n*= 185, 3 years)	SBP = 128 DBP = 79	1 year: SBP = 146 DBP = 86 2 years: SBP = 146 DBP = 87 3 years: SBP = 146 DBP = 85	

* *z*> 2.58 or < −2.58 was considered to be statistically significant (at two-sided α = 0.01). AFREGS, Armed Forces Regression Study; BL, baseline; BP, blood pressure; CABG, coronary artery bypass surgery; CHD, coronary heart disease; CLAS, Cholesterol Lowering Atherosclerosis Study; CME, cholestyramine; CV, cardiovascular; DBP, diastolic blood pressure; EP, end-point; HATS, HDL-Atherosclerosis Treatment Study; HDL-C, high-density lipoprotein cholesterol; MI, myocardial infarction; ns, not significant; SBP, systolic blood pressure; TIA, transient ischemic attack.

In the original Coronary Drug Project (CDP) report, niacin significantly reduced the incidence of definite, non-fatal myocardial infarction over 5–8.5 years of follow-up. No significant changes in BP were found from baseline to end-point (Table 3) (39). However, in a *post hoc* analysis of the CDP in patients with metabolic syndrome according to criteria established by the National Cholesterol Education Program (40), treatment with niacin was associated with a mild but statistically significant reduction in BP compared with placebo at treatment year 1. Compared with baseline, SBP declined by a mean of 2.2 mmHg (vs. +0.8 mmHg with placebo; p < 0.0001), and DBP declined by 2.9 mmHg (vs. −0.9 mmHg with placebo; p < 0.0001) (12).

By categorical analysis of the original CDP data, significantly lower proportions of patients randomised to niacin (vs. placebo) had at least one elevated SBP or DBP reading during 5 years of treatment (excluding patients with abnormal values at baseline). A total of 26.8% of the niacin group had SBP ≥ 160 mmHg compared with 30.6% of the placebo group (*z* = −2.23). A total of 8.7% of the niacin group had SBP ≥ 180 mmHg compared with 10.7% of the placebo group (*z* = −1.77). Corresponding data for DBP were 53.1% of niacin patients having DBP ≥ 90 mmHg compared with 60.4% for placebo (*z* = −3.44) and 6.3% of niacin patients having DBP ≥ 110 mmHg compared with 9.2% for placebo (*z* = −2.84), indicating a significant between-group difference (39).

**Figure 1:**
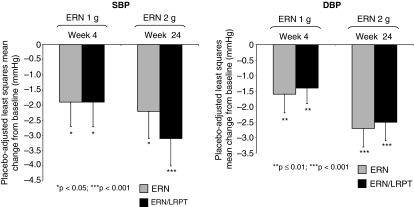
Effects of extended-release niacin alone or with laropiprant on blood pressure. Reductions in blood pressure were significant in patients receiving either extended-release niacin (ERN) or extended-release niacin/laropiprant (ERN/LRPT) compared with placebo at both 4 and 24 weeks (3)

More recent data of large numbers of niacin-treated patients also support these findings. Laropiprant is being investigated as an inhibitor of the prostaglandin D_2_ (PGD_2_) receptor, which mediates flushing (41,42). A short-term titration study of 412 patients administered extended-release niacin 1–2 g/day with laropiprant for up to 8 weeks showed no significant change in BP from baseline (43). However, in a longer (24-week) and much larger (*n*= 1613) study, patients with dyslipidaemia were randomised to one of three treatment arms: extended-release niacin 1 g, extended-release niacin 1 g plus laropiprant 20 mg, or placebo once daily for 4 weeks. Afterwards, the doses were doubled for another 20 weeks of treatment (3). In a *post hoc* analysis of this study, reductions in BP were significant in patients receiving either extended-release niacin or extended-release niacin/laropiprant compared with placebo at both 4 and 24 weeks, and this effect seemed to be dose dependent (Figure 1) (3). Laropiprant neither abolished nor attenuated the BP-lowering effects of niacin, suggesting that the effects of chronic niacin in lowering BP is not because of PGD_2_-mediated flushing.

## Discussion

Clinical trials suggest that infusion of niacin may acutely lower BP in some individuals. Clinical practice experience suggests that, rarely, oral administration may also acutely lower BP, especially when severe niacin-induced flushing occurs. This acute BP lowering is described in the package insert of the currently marketed prescription niacin. It is tempting to speculate that the acute BP effect of acute niacin administration is linked to the vasodilatation induced by niacin. From an efficacy standpoint, niacin activates the G protein-coupled GPR109A receptors on cells such as adipocytes, which may favourably influence lipid metabolism. From an adverse experience standpoint, these same receptors are also found on epidermal Langerhans cells in the skin (44), which generate prostaglandins such as PGD_2_, which in turn, stimulates PGD_2_ type 1 (PD1) receptors on vascular smooth-muscle cells in dermal arterioles, causing vasodilatation and flushing. GPR109A activation on Langerhans cells may also generate PGE_2_ and possibly other mediators that could conceivably influence vascular tone and thus may also contribute to lower BP. Additionally, niacin could conceivably activate its receptor on other cell types (including other components of the cardiovascular system) that might lead to haemodynamic changes. Finally, niacin could have other ‘off-target’ effects (not mediated by GPR109A) that could have acute BP effects.

The potential chronic BP-lowering effects of niacin are best supported by longer niacin trials involving larger numbers of patients. The recent clinical trial experience with laropiprant suggests that any chronic BP-lowering effects of niacin are not likely mediated by DP1 receptors or cutaneous vasodilatation. Thus, the mechanism that might account for chronic lowering of BP by niacin is unknown. One intriguing possibility is that niacin may lower BP at least in part, because of favourable lipid-altering effects that might improve endothelial function. One of the more important lipid effects of niacin is increasing HDL-C levels. HDL particles have many important lipid, antioxidant and anti-inflammatory functions that favourably affect the vasculature. HDL-C levels directly correlate with measures of endothelial function (45). The apolipoprotein B/A-1 ratio is inversely associated with endothelium-dependent vasodilation (46). HDL activates endothelial nitric oxide synthase and generates nitric oxide in endothelial cells in vitro and in vivo (47), and niacin’s HDL-mediated endothelial nitric oxide production may contribute to modest chronic BP lowering with long-term therapy. It is especially interesting to note that, when niacin has been reported to chronically lower BP, the effects have generally been progressive and gradual, occurring over many months or years. These findings correlate to one of the more interesting therapeutic effects of niacin, which is that HDL-C levels may continue to rise, even after many months, and up to a year, after treatment onset (48).

It is somewhat surprising that chronic BP lowering observed with long-term niacin administration, as indicated in studies such as the CDP, has not been more completely evaluated and better characterised, especially given that (i) niacin has been in clinical use for decades; (ii) acute niacin infusion sometimes acutely lowers BP; (iii) multiple clinical trials support the conclusion that niacin favourably affects atherosclerosis; and (iv) elevated BP is a well-established cardiovascular risk factor. As such, the conclusion of this review is limited by the lack of formal BP evaluations in most niacin clinical trials, and a lack of consistent reporting of niacin’s effects upon normotensive compared with hypertensive subjects. It would be of interest to determine whether any demographic or clinical characteristics exist that could help to predict which patients are more likely to experience the haemodynamic effects of niacin, when administered alone or in concert with statins and/or laropiprant to control flushing. Such ongoing trials that might best answer these questions include Atherothrombosis in Metabolic Syndrome with Low HDL/High Triglycerides and Impact on Global Health Outcomes and Heart Protection Study 2–Treatment of HDL to Reduce the Incidence of Vascular Events. But currently, no long-term clinical trial has yet been conducted specifically evaluating niacin’s chronic BP effect as a primary end-point, and controlled clinical trials are needed with ambulatory BP monitoring and other such measures that are more typical for assessing the efficacy of antihypertensive agents. Through such studies and others, a better determination can be made regarding niacin’s effect upon BP, and whether it is possible that the long-term administration of niacin may contribute to its overall cardioprotective benefits.

## Conclusions

Small clinical trials of acute niacin administration have shown significant BP-lowering effects of niacin in patients with hypertension but not necessarily in normotensive individuals. Acute lowering of BP is occasionally found with niacin’s clinical use, and is described in the package insert of prescription niacin. Regarding chronic BP effects, most large, prospective, randomised clinical trials involving niacin and niacin-containing regimens (e.g. the CDP) showed either no clear significant effects of niacin or slightly lower mean BP among some niacin treatment groups compared with placebo. Recent clinical trials involving co-administration of the PGD_2_ receptor antagonist laropiprant suggest that niacin may indeed have dose-dependent chronic BP-lowering effects, which are unlikely to be due to DP1 receptor activation leading to vasodilatation. Future analysis of ongoing niacin clinical trials, and more formalised future clinical trials specifically designed to better assess niacin’s BP effects, may help researchers and clinicians to better appreciate the extent of niacin’s effects on the important cardiovascular risk factor of hypertension.
